# Relationships between Plant Diversity and the Abundance and α-Diversity of Predatory Ground Beetles (Coleoptera: Carabidae) in a Mature Asian Temperate Forest Ecosystem

**DOI:** 10.1371/journal.pone.0082792

**Published:** 2013-12-20

**Authors:** Yi Zou, Weiguo Sang, Fan Bai, Jan Christoph Axmacher

**Affiliations:** 1 UCL Department of Geography, University College London, London, United Kingdom; 2 The State Key Laboratory of Vegetation and Environmental Change, Institute of Botany, Chinese Academy of Sciences, Beijing, China; 3 College of Life and Environmental Science, Minzu University of China, Beijing, China; Tennessee State University, United States of America

## Abstract

A positive relationship between plant diversity and both abundance and diversity of predatory arthropods is postulated by the Enemies Hypothesis, a central ecological top-down control hypothesis. It has been supported by experimental studies and investigations of agricultural and grassland ecosystems, while evidence from more complex mature forest ecosystems is limited. Our study was conducted on Changbai Mountain in one of the last remaining large pristine temperate forest environments in China. We used predatory ground beetles (Coleoptera: Carabidae) as target taxon to establish the relationship between phytodiversity and their activity abundance and diversity. Results showed that elevation was the only variable included in both models predicting carabid activity abundance and α-diversity. Shrub diversity was negatively and herb diversity positively correlated with beetle abundance, while shrub diversity was positively correlated with beetle α-diversity. Within the different forest types, a negative relationship between plant diversity and carabid activity abundance was observed, which stands in direct contrast to the Enemies Hypothesis. Furthermore, plant species density did not predict carabid α-diversity. In addition, the density of herbs, which is commonly believed to influence carabid movement, had little impact on the beetle activity abundance recorded on Changbai Mountain. Our study indicates that in a relatively large and heterogeneous mature forest area, relationships between plant and carabid diversity are driven by variations in environmental factors linked with altitudinal change. In addition, traditional top-down control theories that are suitable in explaining diversity patterns in ecosystems of low diversity appear to play a much less pronounced role in highly complex forest ecosystems.

## Introduction

Terrestrial arthropods play important roles in ecosystem functioning, for example in pollination, pest control and in occupying key positions in carbon and nutrient cycling through food-web links. These roles also strongly impact on plant diversity patterns [1]–[4]. Simultaneously, plant species richness is also believed to affect diversity and abundance of arthropods throughout the trophic chain via bottom-up effects [5]. An increase in plant diversity can generally enhance net primary productivity [6], [7], which in term provides more food resources for herbivorous arthropods, hence increasing the overall biomass of arthropod consumers [8], [9]. Apart from this control via food source effects, arthropod consumers are also known to be influenced by top-down control via the abundance of their natural enemies [10]–[12]. This control forms the basis of the “Enemies Hypothesis” [13], which postulates that species-rich vegetation assemblages can provide more refuges and more stable prey availability for predators than plant species-poor assemblages, resulting in predators catching and feeding on prey more effectively, so that a higher diversity in the plant community is believed to support a higher diversity and abundance also of predatory species [11], [14].

A positive association between phytodiversity and both diversity and abundance of herbivorous arthropods has been found in a variety of ecological experiments [4], [5], [9], [15], in low-diversity grassland [16]–[18] and in agriculture fields [19], [20]. Nonetheless, reports of negative relationships between plant diversity and the diversity of arthropod taxa are also common, backed again with results from experiments [21], grassland ecosystems [22], [23] and agricultural landscapes [24]. In complex forest ecosystems, a number of studies report a positive feedback between the diversity of plants and herbivorous insects [25]–[27]. Other studies nonetheless also report a lack of significant relationships [28], [29] or even negative correlations [30], [31].

For studies investigating links between the phytodiversity and the diversity and abundance of predatory arthropods, the Enemies Hypothesis has been supported by a range of experimental studies [5], [32]–[34] and by studies in agricultural [19], [35] and grassland [36] ecosystems with relatively low phytodiversity levels. It is predicted that top-down control of herbivores by natural enemies would be more predominant in a perennial ecosystem than annual ones due not least to the more consistent prey availability [21], [37]. The associated positive link between plant diversity and the diversity and abundance of predatory arthropods is therefore predicted to be stronger in natural forest ecosystems in comparison to annual grassland and agriculture fields. Nonetheless, very little research has been conducted to date into these links in the world’s remaining mature forest ecosystems. The limited published data suggests that areas of high phytodiversity do not automatically support a high diversity in predatory arthropods [38]. The underlying patterns are not yet well-understood, and more studies into the links between the vegetation and predatory arthropod taxa in natural forest ecosystems are urgently needed [39], [40].

Among important predatory arthropod taxa, ground beetles (Coleoptera: Carabidae) are one of the most species-rich families, comprising more than 40,000 described species [41]. Members of this family, which is chiefly composed of true predators and omnivorous species, have been widely used in ecological studies due to their environmental sensitivity and the good knowledge base existing in relation to their taxonomy and ecology [41]–[46]. Ground beetles are believed to generally benefit from high levels of plant diversity [47], and their abundance and diversity is believed to directly impact on ecosystem functioning [41], [48], [49].

In our study, we used ground beetles as target group to analyse the relationship between abundance and diversity of predatory arthropods and phytodiversity. The study was undertaken in one of the largest remaining mature temperate forest ecosystems in China. To our knowledge, this is the first study specifically focussing on diversity relationship between plants and predatory arthropods in species-rich, mature temperate forests in Asia. Our objectives were 1) to test if positive links between ground beetle abundance and diversity and the diversity in plant species exist as predicted by the Enemies Hypothesis, and 2) to establish how environmental factors affect the observed links.

## Materials and Methods

### Ethics

This study was carried out in Changbaishan Nature Reserve and all samplings were permitted by the Changbaishan Nature Reserve Management Center. The field study did not involve any endangered or protected species.

### Study Area

Our study area is located on the northern slopes of Changbai Mountain (E 127°43′ –128°16′; N 41°41′ –42°51′) in Jilin Province, North-eastern China. The local climate is influenced by summer monsoon rains. The study area experiences dry, windy spring conditions followed by a short, wet summer, a cool autumn with widespread fog and a long, cold winter [50]. The pristine forest vegetation is composed of evergreen and deciduous coniferous and broad-leaved tree species. The forests on Changbai Mountain follow a distinctive altitudinal zonation, with a mixed coniferous and broad-leaved forest zone below 1100 m, a mixed coniferous forest zone between 1100 and 1500 m and a sub-alpine mixed coniferous forest zone between 1500 and 1800 m. The upper forest boundary is composed of birch forests reaching elevations of up to 2100 m, followed by a tundra zone with dominating dwarf trees and shrub formations [51].

We selected a total of 33 plots between altitudes of 700 m and 2000 m, representing all aforementioned forest types (see details in [52] ). Of these, 31 plots were sampled both in 2011 and 2012. To increase sampling intensity in the birch forest where overall beetle abundance was very low, two additional birch forest plots were sampled in 2012.

### Vegetation Survey and Carabids Sampling

Each study plot had a size of 20×20 m^2^ and divided equally into four subplots. In the centre of each sub-plot, a pitfall trap was placed. For the recording of the vegetation, the entire 400 m^2^ plot was divided into 4 sub-plots, and all trees and shrubs were recorded in each of the resulting sub-plots. Herbaceous species were recorded in four plots of 1 m^2^ that were randomly located within each of the sub-plots. The number of individuals was recorded for each plant species in each layer. The breast height area was recorded for each tree specimen and the average height was recorded for each shrub and herb species.

The pitfall traps for carabid sampling consisted of a 250 ml plastic cup with an open diameter of 7.5 cm. To minimize the attractant bias, we used saturated salt (NaCl) water as killing solution and to preserve specimens in the sampling period [53]. Each cup was filled to about half its volume with saturated salt water, with detergent added to break the water surface tension. Above each trap, a solid aluminium roof of 10×10 cm^2^ was placed to protect the solution from dilution and litter contamination. Carabids were continually sampled from early July to early August in 2011 and from late June to late August in 2012, with traps routinely emptied and refilled in about 10 day-intervals. As it was impossible to sample all plots on the same day due to the large geographical area and other logistical reasons, sampling days varied between 22 and 34 for the different plots in 2011, and between 42 and 65 days in 2012.

### Data Analysis

Carabid activity abundance of each plot was calculated as the overall number of sampled individuals divided by the total sampling period in days, resulting in a mean daily catching rate for each plot. Values were calculated for the entire sampling period in 2011 and 2012 to minimize effects of inter-annual variations. Carabid α-diversity was measured as Fisher’s α, a widely used parametric index considered robust in measuring arthropod diversity of samples varying in sample size [28], [31], [54], [55]. Shannon diversity of the vegetation was calculated individually for each plant layer based on the important value (IV) of each plant species to avoid the bias from simple abundance-based calculations [56], [57]. The IV contains three aspects reflecting the relative contributions of each plant species towards each layer: relative abundance (*d*), relative frequency (*f*) and relative dominance (*h*). The IV for *i* ^th^ species in *j* ^th^ sample plot is calculated as:
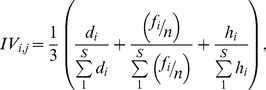
where *d* is the number of individuals, *f* is the number of subplots in which the species occurred, *n* is the total number of subplots in a sample plot (4 in this case), and *h* is the breast height area for tree species and the height for shrub and herb species.

Our modelling of plant-carabid relationships was based on multiple linear regressions, where carabid activity abundance and α-diversity were used as response variables, respectively. A series of stepwise linear regression analyses was performed to identify the most important independent variables. Stepwise selection was used with both forward selection for selection of variables contributing significantly (*P* = 0.05) towards the model and backward elimination to verify that variables made no significant contribution in the selection of new variables. Vegetation variables included the total number of plant species (PS), the Shannon diversity (H) for trees (TH), shrubs (SH) and herbs (HH), and the abundance density (D) for trees (TD), shrubs (SD) and herbs (HD), respectively. Modelling included all vegetation parameters as independent variables first and then added elevation as additional independent variable.

To account for the substantial forest vegetation changes with changing elevation, we used Principal Components Analysis (PCA) based on the presence-absence data of plant species to establish the existence of distinct sample clusters representing a relatively homogeneous vegetation composition and re-ran the linear regression models separately for the different clusters.

All statistical analysis was carried out in R [58], using the packages “spaa” [59] to calculate the Shannon diversity index and “Vegan” [60] to carry out the PCA and to calculate Fisher’s α values.

## Results

We recorded a total of 178 plant species belonging to 128 genera and 58 families. The tree layer was comprised of 32 species belonging to 20 genera and 12 families; the shrub layer contained 43 species of 28 genera and 15 families and the herb layer comprised 112 species representing 88 genera and 43 families. Pitfall traps caught 4844 carabids. Ten specimens (0.2%) were not identified due to substantial damage. The remaining 4834 individuals were separated into 34 species and 13 morphospecies. A detailed species list has been provided in [52]. The overall average daily activity abundance for the entire study area was 1.83 individuals per plot.

### Carabid-plant Relationships

Stepwise regression entering all vegetation parameters produced two subsequent models predicting carabid activity abundance. The first model (adjusted R^2^ = 0.43, F_1,31_ = 24.63, *P*<0.001) included SH as significant negative (β = −0.925, *P*<0.001) predictor of carabid abundance, while the second model (adjusted R^2^ = 0.51, F_2,30_ = 17.29, *P*<0.001) additionally included HH as positively (β = 0.427, *P* = 0.02) affecting beetle abundance. The Akaike information criterion (AIC) slightly decreased from 79.35 to 75.34 (Table 1, Models 1 and 2). The model predicting carabid α-diversity again included SH as main predictor (β = 0.571, *P* = 0.039), which in this case was positively correlated with Fisher’s α values. Overall, this model performed not as well (Table 1, Model 4, adjusted R^2^ = 0.10, F_1,31_ = 4.65, AIC = 102.45). When elevation was entered as additional independent variable, MLR models for both, carabid activity abundance and α-diversity, only included this parameter as significant, with model fits markedly improved (AIC = 71.71, adjusted R^2^ = 0.54, F_1,31_ = 39.13, beta = 1.753, *P*<0.001, and AIC = 96.27, adjusted R^2^ = 0.26, F_1,31_ = 12.11, beta = −1.451, *P* = 0.002, respectively, Table 1, Models 3 and 5). Models therefore predict an increased beetle abundance at a reduced diversity with increasing elevation.

**Table 1:** Stepwise linear regression models using activity abundance and Fisher’s α-diversity of carabids as dependent variables, respectively, only using vegetation parameters as independent variables (Model 1, 2 and 4) and including elevation as additional independent variable (Model 3 and 5).

Dependent variable	ModelNo.	Adjusted R^2^	F	ModelP-value	Model AIC	Selected independent variable(s)	β	Std. Error of β	t	P-value
Activity abundance	1	0.43	24.63	<0.001	79.35	SH	−0.925	0.186	−4.96	<0.001
	2	0.51	17.29	<0.001	75.34	SH	−0.897	0.173	−5.17	<0.001
						HH	0.427	0.174	2.45	0.02
	3	0.54	39.13	<0.001	71.71	ASL(km)	1.753	0.280	6.26	<0.001
Fisher’s α-diversity	4	0.1	4.65	0.039	102.54	SH	0.571	0.265	2.16	0.039
	5	0.26	12.11	0.002	96.27	ASL(km)	−1.415	0.530	−3.48	0.002

TH: Shannon diversity for trees; SH: Shannon diversity for shurbs: TD: the abundance density for trees; SD: the abundance density for shrubs; Low: low elevation zone of less than 1000 m; Middle: intermediate elevation zone of 1000–1500 m; High: high elevation zone of 1500–2000 m.

### Carabid-plant Relationship in the Different Vegetation Types

**Figure 1:**
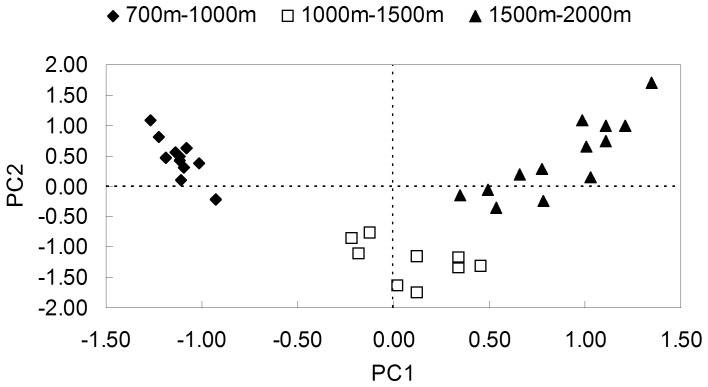
PCA ordination plot based on vegetation composition showing three distinct clusters (proportion variance explained for PC1 = 24% and for PC2 = 11%; eigenvalues for PC1 = 4.83 and PC2 = 2.12).

The ordination plot of the first two principle components (PCs) based on vegetation composition showed three distinctive clusters along the elevational gradient (Figure 1). A first cluster of eleven plots represents the low elevation zone below 1000 m covered by mixed coniferous and broad-leaved forests, while the second cluster consists of 9 plots located in a median elevation zone between 1000 m and 1500 m, containing 3 plots within the mixed coniferous and broad-leaved forest type and 6 plots within mixed coniferous forests, and the cluster of the remaining 13 plots represents the high elevation zone above 1500 m covered by sub-alpine mixed coniferous forests and birch forests.

Below 1000 m in the mixed coniferous and broad-leaved forest elevation zone, the first model using vegetation parameters as independent variables indicated that carabid activity abundance was negatively associated with TH (β = −1.67, *P* = 0.002) (Table 2, Model 6, AIC = 5.37, adjusted R^2^ = 0.62, F_2,8_ = 17.37, *P* = 0.002), while the second model also included a positive relationship with SD (β = 0.012, *P* = 0.012), with a highly significant overall model fit (Table 2, Model 7, AIC = −1.79, adjusted R^2^ = 0.82, F_2,8_ = 23.07, *P*<0.001). At the intermediate elevation zone, only TD (β = 0.002, *P* = 0.011) was included in predicting carabid activity abundance (Table 2, Model 8, AIC = 14.12, adjusted R^2^ = 0.57, F_1,7_ = 11.65, *P* = 0.011). For the highest elevation zone, SH (β = −1.435, *P*<0.001) was the only independent variable included in the model (Table 2, Model 9, AIC = 21.13, adjusted R^2^ = 0.73, F_1,11_ = 32.65, *P*<0.001).

**Table 2:** Results of stepwise linear regressions for the three elevational zones using activity abundance and Fisher’s α-diversity of carabids as dependent variables and vegetation parameters as independent variables.

Dependent variable	Elevation zone	Model No.	Adjusted R^2^	F	ModelP-value	Model AIC	Selected independent variable(s)	β	Std. Error of β	t	P-value
Activity abundance	Low	6	0.62	17.37	0.002	5.37	TH	−1.670	0.401	−4.17	0.002
		7	0.82	23.07	<0.001	−1.79	TH	−1.236	0.310	−3.99	0.004
							SD	0.012	0.004	3.24	0.012
	Middle	8	0.57	11.65	0.011	14.12	TD	0.012	0.003	3.41	0.011
	High	9	0.73	32.65	<0.001	21.13	SH	−1.435	0.251	−5.71	<0.001
Fisher’s α-diversity	Low	10	0.40	7.74	0.021	21.79	SD	−0.058	0.021	−2.78	0.021
	High	11	0.27	5.37	0.041	22.41	TD	−0.004	0.157	−2.32	0.041

TH: Shannon diversity for trees; SH: Shannon diversity for shurbs: TD: the abundance density for trees; SD: the abundance density for shrubs; Low: low elevation zone of less than 1000 m; Middle: intermediate elevation zone of 1000–1500 m; High: high elevation zone of 1500–2000 m.

None of the vegetation diversity variables was significantly linked with carabid α-diversity in any of the three distinct elevation zones. Nonetheless, carabid α-diversity was linked to the vegetation density parameters SD (β = −0.058, *P* = 0.021) at the low elevation zone (Table 2, Model 10, AIC = 21.79, adjusted R^2^ = 0.40, F_1,11_ = 7.74, *P* = 0.021) and TD (β = −0.004, *P* = 0.041) at the high elevation zone (Table 2, Model 11, AIC = 22.41, adjusted R^2^ = 0.27, F_1,11_ = 5.37, *P* = 0.041). Neither herb diversity nor herb density appears to be linked to either activity abundance or α-diversity of carabids at any of the three elevation zones.

## Discussion

Firstly, our study underlines that changes in elevation were the predominant drivers of changes in both, carabid abundance and α diversity patterns in CNR, while the overall phytodiversity was not significantly correlated with either abundance or α-diversity of the beetles. Parameters associated with altitudinal changes like temperature and precipitation are therefore more important in influencing the diversity of ground beetles than plant diversity *per se*, which is also consistent with findings for herbivorous insect diversity patterns [28], [29], [61], [62]. Accordingly, we believe that the observed relationships between activity abundance and α-diversity of carabids and vegetation variables recorded for the entire elevational gradient are mainly driven by the underlying changes in the environmental factors.

Nonetheless, the observed, highly significant negative correlation between carabid activity abundance and the diversity of shrubs stands in direct contradiction to the Enemies Hypothesis, which predicts a positive relationship with carabid abundance. Our observations also stand in contrast to most studies conducted in agricultural [19], [35] and grassland ecosystems [36], which differ from our study site by their markedly lower overall phytodiversity. Similar to us, Koricheva et al. [63] also reported a negative relationship between plant diversity and activity abundance of predatory arthropods in a grassland ecosystem. One of the explanations they present for this negative trend was a reduction in predator activity density with an increase in herb density, but this trend was not supported by our investigations. However, our results are consistent with observations by Schuldt et al. [38] who observed that activity abundance of spiders was also reduced in areas with an increased woody plant diversity in a natural forest in Zhejiang Province. Schuldt et al. [64] and Vehviläinen et al. [65] state that the abundance of predatory species depends more strongly on the presence of specific tree species rather than on overall tree diversity. The lack of validity of the Enemies Hypothesis for complex forest ecosystems might therefore relate to the multifaceted interactions between specific plant species, their herbivores and the predatory insect assemblages inhabiting these ecosystems [39], [66]. Although we did not investigate the role of specific tree or shrub species and their functional groups, an increase in woody plant diversity can be linked to either, an increase in their evenness or their species richness, and hence might potentially reflect a reduction in the overall dominance of specific favourable species, which could explain the reduction in carabid abundance. Alternatively, a high plant diversity can potentially support a higher density of herbivorous arthropods in natural forests [25]–[27], [67], which might also result in a reduction of predators’ overall foraging time and hence their recorded activity density [38]. An increased plant diversity and the associated assumed increase in herbivores can furthermore provide an increase in food sources and niches for competing predatory arthropod taxa such as spiders and ants, increasing the overall competition levels for prey and consequently reducing the overall abundance of carabids. As food chains and habitats are much more complex in forests in comparison to most other terrestrial ecosystems [68], traditional top-down control theories that are suitable in less heterogeneous ecosystems may overall be difficult to apply here [39].

The reported positive relationship between carabid activity abundance and woody plant density at low and intermediate elevation zones potentially reflects a bottom-up effect: plots with a high density in woody plants are likely to be very productive. High woody plant density can not only enhance shading and soil moisture levels and hence create favourable microhabitats for carabids and their larvae [69], but also producing more leaf litter, which can in term improve soil fertility and increase food availability for carabids [70], [71]. The negative correlations between woody plant density and the α-diversity of carabids within low and high elevation forest communities might again relate to changes in arthropod diversity being related to changes in the woody plant species composition, rather than their overall diversity [23], [72]. Additionally, impacts of plant diversity are known to decrease with increasing trophic levels [5], [73], so that impacts of the vegetation on the diversity of predatory insects is often quite complex.

The negative relationship between altitude and the α-diversity of carabids can be explained by the Harsh Environmental Hypothesis. Accordingly, species at high altitudes experience harsh climatic conditions requiring them to have broader overall tolerance ranges than species at low altitudes, which in term leads to wider distribution ranges with increasing elevation and to a higher species richness at low altitudes [74]–[76]. This hypothesis is supported by the observed increase in carabid species’ altitudinal ranges with increasing elevation that we previously reported [52]. A possible reason for the positive links between carabid activity abundance and elevation could be the reduction of competitors such as ants and other predatory arthropods [77] or changes in the activity patterns due to potential scarcity of food resources, which would also increase the number of specimens sampled at high altitudes.

Our results finally showed that the density of herbaceous plants did not significantly influence carabid activity abundance nor their diversity, which stands in strong contrast to studies in grassland ecosystems that commonly record negative relationships [78]–[80]. Pitfall traps have been widely used in surveys of ground-dwelling arthropods [42], [78], [81]–[84] and can be considered as a standard method in ground beetle sampling [42]. Nonetheless, one of the main known pitfalls of pitfall trapping is the dependency of the sampling rate on both the target population density and the individual specimen’s activity [78], [85]–[87]. Factors affecting this activity need to be taken into consideration when comparing pitfall samples, and it has commonly been suggested that vegetation density particularly of herbaceous species needs to be considered in the respective data interpretation [79], [80]. The negative impact of this density in grassland ecosystems is believed to be due to a reduction in the ground beetle mobility caused directly by a dense herb layer [79], [80]. However, our results strongly suggest that the density of this layer in the old-growth forests on Changbai Mountain is not dense enough to significantly affect carabids’ movements, supporting the argument that the influence of the density in understory vegetation can be neglected when studying forest carabid assemblages [38]. Nonetheless, controlled capture-recapture experiments would be needed to evaluate the exact effects which might be present.

Overall, our results clearly indicate that, in highly complex forest ecosystems, predatory arthropod abundance and diversity patterns do not support traditional top-down control theories that are suitable for less complex ecosystems. To substantiate these conclusions and establish if the Enemies Hypothesis is generally unsuitable for complex forest ecosystem, we suggest long-term monitoring of a wider range of predatory arthropod groups (e.g. spiders, ants and centipedes) not only in temperate, but also in tropical and subtropical forests where the complexity of food-webs is even greater. Other temperate mature forests in Northeast China, such as Liangshui and Fenglin Natural Reserve in Heilongjiang Province, are ideal study areas to substantiate results from species-rich temperate forests. These latter studies in some of the last remaining highly diverse mature temperate forest ecosystems in NE China would also allow a better understanding of the complex inter-linkages between taxa and across trophic levels, with particular foci on the role of the woody plant species composition on predator distribution patterns and on the mechanisms governing responses of herbivorous arthropods to changes in plant diversity and species composition.
